# Unusual Case of Extensor Indicis Attachment: Cadaveric Observation and Clinical Relevance

**DOI:** 10.7759/cureus.103781

**Published:** 2026-02-17

**Authors:** Donovan B Turpin, Garrett T Folds, Caleb T Lee, Alexandra G Nolen, Andrew M Schwartz, Adegbenro Fakoya, Hosne Ara

**Affiliations:** 1 School of Medicine, Louisiana State University Health Sciences Center, Shreveport, USA; 2 Department of Cellular Biology and Anatomy, Louisiana State University Health Sciences Center, Shreveport, USA

**Keywords:** anatomical anomaly, dorsum of hand, extensor indicis, muscle attachment, muscle origin

## Abstract

Variations in anatomical structure present challenges to patients as well as medical professionals. In reference to patients, it could affect their lifestyle in terms of function. As for the medical professionals, treatments such as surgery could be made more difficult. Knowing the rare variations leads to better medical care and patient satisfaction and prevents unexpected complications. We discuss the extensor indicis (EI) muscle and the extensor indicis brevis (EIB), an anatomical variation we found while dissecting. The EIB had an irregular origin and insertion. We will further discuss and explore the implications of the variation.

## Introduction

The extensor indicis (EI) is a slender, elongated muscle of the posterior compartment of the forearm that plays a crucial role in extension of the second digit. It is present in most individuals, with cadaveric studies estimating its prevalence at 96.5% [1]. Usually, it originates from the proximal two-thirds of the distal half of the interosseous line of the ulna, the adjacent interosseous membrane, or from fibrous septa associated with the extensor pollicis longus and the dorsal ulnar septum. The muscle fibers of the EI converge to form a tendon that courses distally through the fourth extensor compartment of the wrist, deep to the radial tendons of the extensor digitorum communis (EDC). The tendon crosses the wrist and inserts into the extensor expansion of the index finger, ulnar to the corresponding common extensor tendon [2]. The EI is innervated by the posterior interosseous nerve, a branch of the radial nerve (roots C7-C8), and functions to extend the index finger while assisting in wrist extension [3].

Anatomical variations of the EI have been extensively documented. In reference to its atypical variants, the normal EI is referred to as extensor indicis proprius (EIP). A varied EI can have multiple tendon heads, including variants with two slips (7.2%) and three slips (0.3%) [1]. In some cases, the EI tendon splits and connects to both the index finger and other digits, such as the thumb (0.75%), middle (1.6%), and ring fingers [1,4]. The EI tendon can also insert directly into the middle finger (3.7%) instead of the index finger [1]. Variations of the EI muscle can be relatively small (3%) and can have abnormal origins [2].

**Figure 1:**
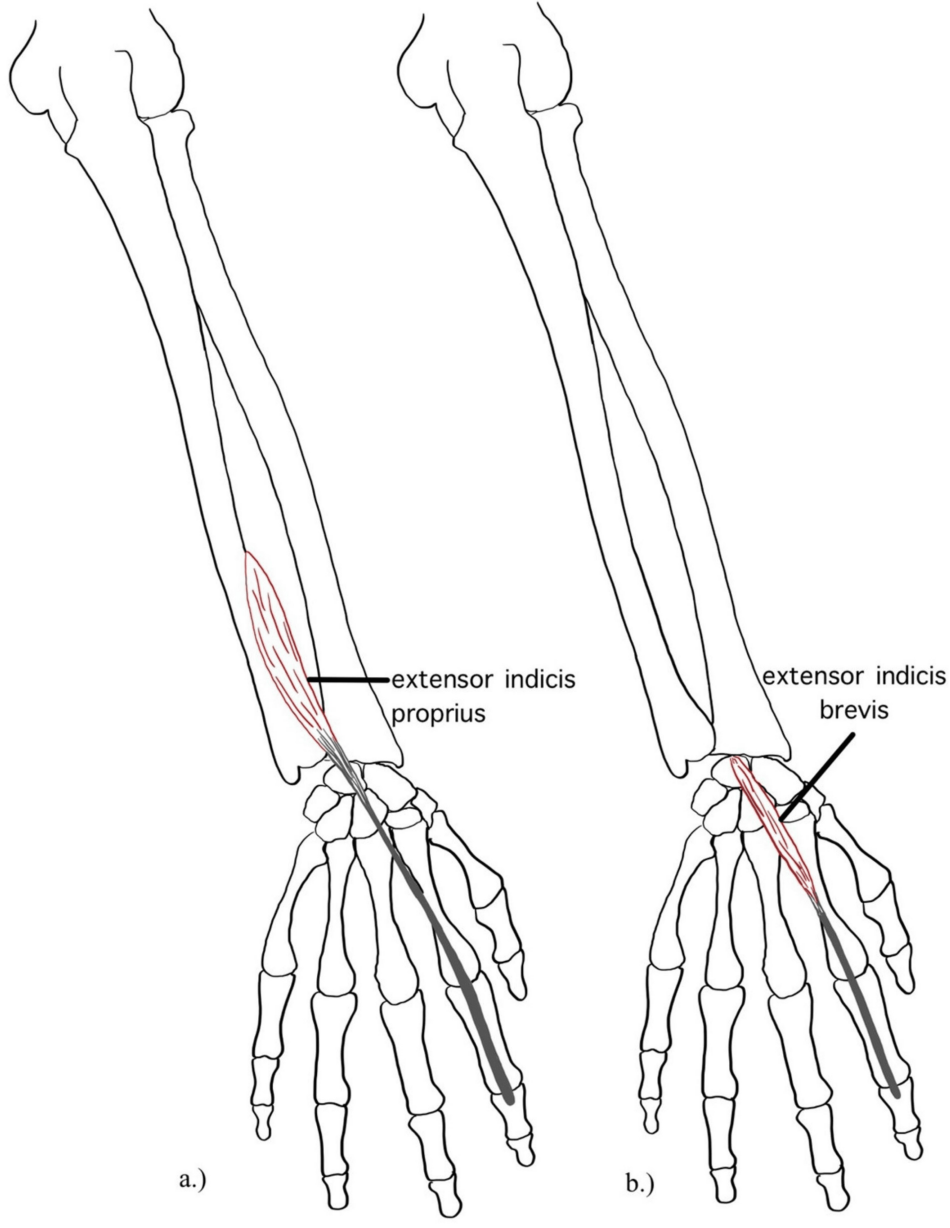
a) Illustration of extensor indicis proprius. b) Illustration of extensor indicis brevis, an anatomical variation of extensor indicis.

One such abnormal variant of the EI is the extensor digitorum brevis manus (EDBM). It is described as a short extensor with an origin in the dorsal carpal region and insertion into the index or middle fingers [4]. EDBM has a prevalence of 4% and is bilateral in 26% of cases [1]. Bernhard Siegfried Albinus first reported this variant in 1758, and it has since been classified into three categories [5,6]. EDBM type I, which is also known as extensor indicis brevis (EIB), inserts into the index finger with no accessory EI tendon present (see Figure 1). EIB makes up 31% of EDBM cases [1,6,7]. EDBM type II (46% of EDBM cases) can be divided into three subtypes, each of which includes EDBM and accessory EI tendons inserting into the index finger [6]. EDBM type III inserts into the middle finger with or without an accessory EI and makes up 23% of EDBM cases [1,6].

In this report, we describe a rare case of bilateral EIB and examine possible clinical significance. The aim of this study is to present a rare case of EIB and to explore the anatomical variations and the clinical significance of the location of the EI muscle.

## Case presentation

Medical students at Louisiana State University Health Sciences Center in Shreveport underwent routine cadaveric dissection of a 90-year-old white female, fixed in formalin. During the dissection of the posterior forearm, the students noticed the absence of the EIP bilaterally from its conventional setting in the posterior antebrachial compartment. However, upon further dissection at the dorsum of the hand, an EIB was found to be present bilaterally.

**Figure 2:**
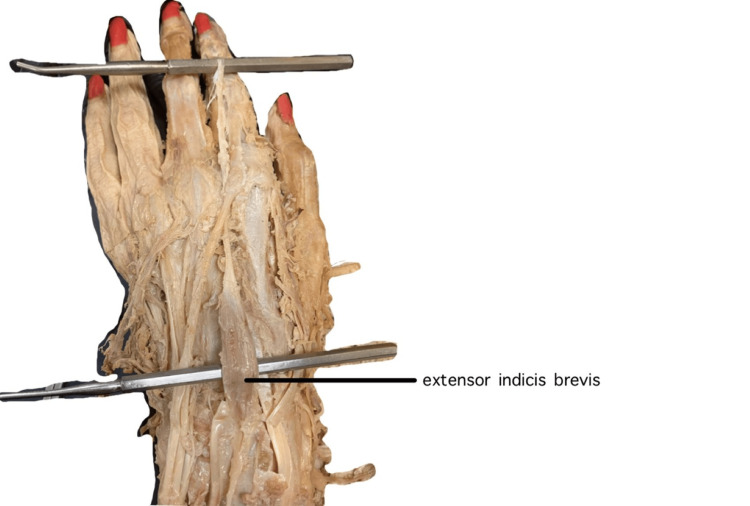
Dorsal view of extensor indicis brevis originating from radial carpal bones and inserting into extensor hood, elevated by the probes.

Upon further inspection, the EIB appeared to originate from radial carpal bones proximally in the wrist joint. In tracing the tendon toward the index finger, it passed deep to the larger EDC tendon, inserting ulnar to it in the extensor expansion. The tendon continued to run parallel to the EDC tendon even to the middle and distal phalanges (Figures 1-2).

**Figure 3:**
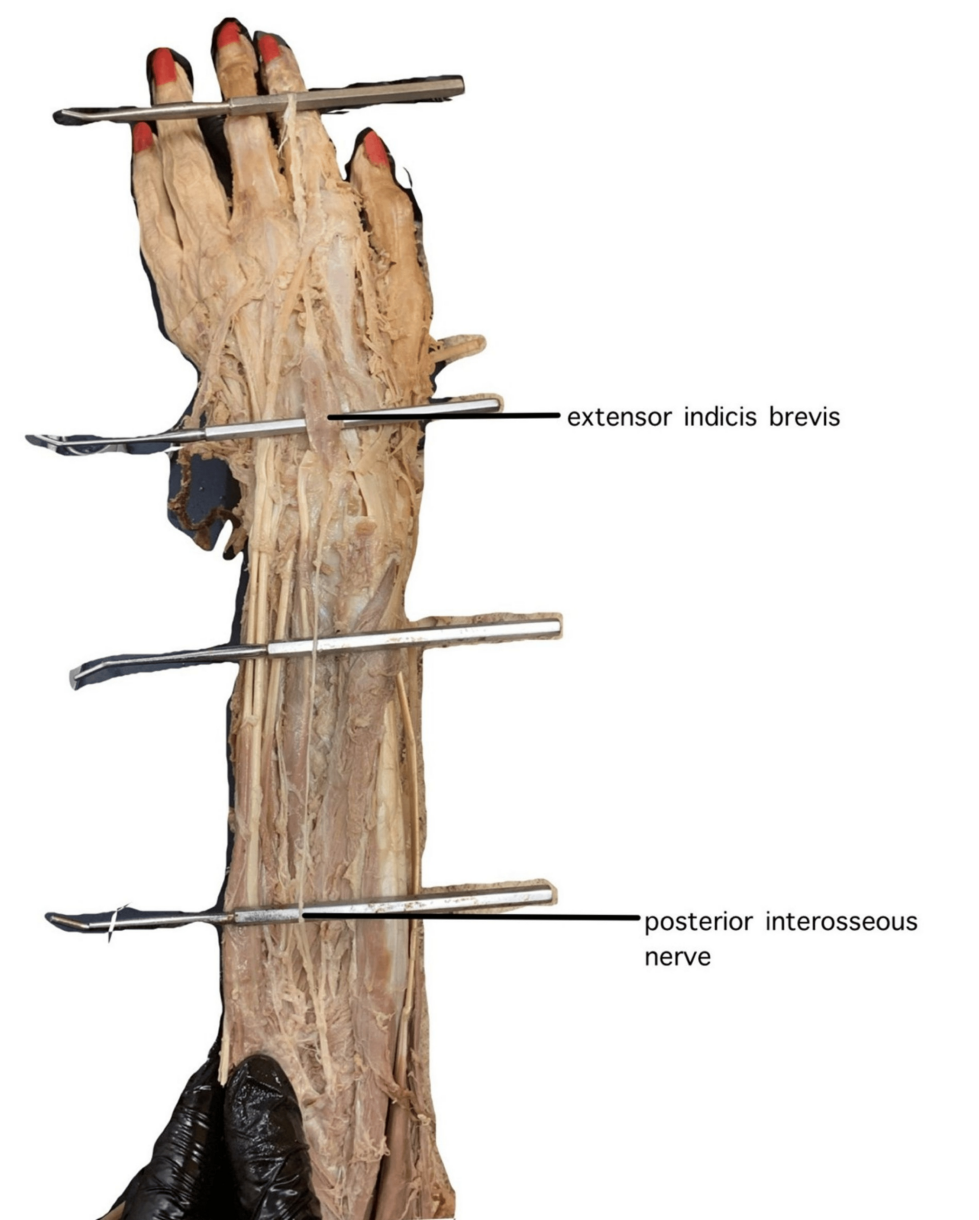
Dorsal view of extensor indicis brevis and the posterior interosseous nerve, both originating from radial carpal bones, elevated by the probes.

The innervation is most likely the posterior interosseous nerve, the same as many muscles of the posterior antebrachial compartment. Upon tracing the nerve from where it pierces the supinator muscle, it was found to travel past the wrist joint to the origin of the EIB (Figure 3). The presumed blood supply would come from the radial artery via the basal metacarpal arch.

## Discussion

Fortuitous discovery of an EIB in the setting of cadaveric dissection gives an opportunity to discuss its importance for surgical and clinical settings. Accurate diagnosis is important to prevent misidentification as a pathologic structure, such as a ganglion cyst or other soft tissue mass [1,8]. Misdiagnosis or failure to recognize these anatomical variants can lead to unnecessary interventions, delayed treatment, or missed opportunities for more effective therapeutic strategies [1].

EIB and other EDBM variations are exceedingly rare clinically, and most cases are likely asymptomatic. However, it is possible that EIB can alter grip strength and dexterity of the affected hand, which may also play a role in a physical therapist’s evaluation and planning during rehabilitation [9,10]. EIB may also be relevant in surgical procedures such as tendon transfers or reconstructive hand surgery, where knowledge of these variations could help avoid complications [9,11]. Moreover, EIB may potentially be implicated in peripheral nerve compression disorders, along with wrist pain from swelling or inflammation [12,13].

Limitations of the study include the lack of availability of certain demographics and other measures (i.e., ethnicity and muscle/tendon measurements).

## Conclusions

The EIB variant observed in this study represents a rare bilateral anatomical variation, with notable absence of the traditional EIP. Although often asymptomatic, such variants may have clinical consequences, including potential misdiagnosis as soft tissue masses, contribution to dorsal wrist pain, or complications during tendon transfers and dorsal wrist surgeries. Awareness of this variation is important for clinicians to avoid diagnostic confusion and optimize surgical outcomes.
